# Circulating muscle- and inflammation-related microRNAs in breast cancer survivorship: associations with subtype, treatment, timing, and age

**DOI:** 10.1007/s11033-026-12472-9

**Published:** 2026-07-28

**Authors:** Yanping Jiang, Heidi Annuk, Nicola Miller, Kerin Michael, Sai Zhang, Sanjeev Gupta, Ananya Gupta

**Affiliations:** 1 Discipline of Physiology, University of Galway, Galway, H91 TK33 Ireland; 2 Discipline of Surgery, University of Galway, Galway, H91 TK33 Ireland; 3 Discipline of Pathology, University of Galway, Galway, H91 TK33 Ireland; 4 Scientific Research Center of Xiangya Hospital, Central South University, Changsha, 410008 China

**Keywords:** miRNA, Muscle, Inflammation, Breast cancer, Survivorship

## Abstract

**Purpose:**

Cancer and its treatments often lead to inflammation, muscle problems, and ongoing loss of physical function, as reflected by alterations in circulating microRNAs (miRNAs). In this study, we measured levels of selected muscle- (miR-1, miR-133, miR-208, miR-486, and miR-499) and inflammation-related (miR-21, miR-126, miR-146, and miR-155) miRNAs in breast cancer patients and aimed to explore, using stratified analyses, how their levels relate to cancer subtype, treatment modality, treatment timing, and age.

**Methods:**

We collected 77 plasma samples from pretreatment and post-treatment breast cancer patients and healthy controls. Circulating miRNA levels were measured using quantitative reverse transcription polymerase chain reaction. We compared miRNA levels across different breast cancer subtypes, treatment modalities, treatment timing, and age groups.

**Results:**

Pretreatment patients with breast cancer did not show significant changes in the selected miRNAs compared with healthy controls. However, different breast cancer subtypes showed distinct patterns: Luminal A predominantly affected muscle-related miRNAs, and Luminal B affected inflammation-related miRNAs. Cancer treatment, especially surgery and chemotherapy, led to significant changes in miR-21 and miR-486, primarily within the first 91 days after cancer diagnosis. Increases in miR-133 and miR-486 after treatment were mostly seen in patients over 50 years old.

**Conclusion:**

Circulating muscle- and inflammation-related miRNAs display distinct expression patterns associated with breast cancer subtype and treatment. Specifically, miR-21, miR-133, and miR-486 demonstrate sensitivity to cancer treatment exposure, timing, and patient age.

**Supplementary Information:**

The online version contains supplementary material available at 10.1007/s11033-026-12472-9.

## Introduction

In 2022, breast cancer was the most frequently diagnosed malignancy among women, with more than 2.3 million cases. Advances in multimodal therapy have increased the chance of cure in 70–80% of patients [1, 2]. However, cancer- and treatment-induced complications can exert negative effects on treatment efficacy and even significantly increase morbidity and mortality. Sarcopenia refers to the loss of muscle mass and strength and is one of the most severe complications that breast cancer survivors face during and after treatment. Studies have shown that the prevalence of sarcopenia ranges from 30 to 55% in breast cancer patients, and it is linked to a higher chance of chemotoxicity, functional decline, and poorer quality of life during survivorship [3]. Sarcopenic survivors have a 2.86-fold greater all-cause mortality compared to their non-sarcopenic counterparts [4]. The mechanisms underlying cancer- and treatment-induced sarcopenia are complex, and one of these mechanisms may be involved in inflammation [5, 6]. Inflammation can overstimulate the immune system and increase energy expenditure, thereby depleting stored reserves and affecting general metabolism. Such a dynamic shift may result in skeletal homeostasis toward muscle loss [7].

MicroRNAs (miRNAs) are short non-coding RNAs, with 20–22 nucleotides in length, and evidence has demonstrated that miRNAs play an important role in modulating pathways related to skeletal muscle turnover and inflammation [8, 9]. For example, Narasimhan et al. [10] found eight upregulated miRNAs in patients with cancer cachexia compared with their non-cachectic counterparts by next-generation sequencing. Ingenuity pathway analysis showed that the targets of these miRNAs were known to engage in myogenesis, inflammation, and immune response. These results indicated that miRNA may directly or indirectly be involved in muscle development and function, and the abnormal expression of miRNAs may imply muscle loss and dysfunction during cancer survivorship. While research has shown that exercise can restore muscle loss and strength by improving systemic inflammation, this process is accompanied by alterations in miRNAs. In our previously published review, we summarized the role of miRNAs in both cancer- or cancer treatment-induced muscle loss and exercise. Generally, cancer and its treatment can cause muscle damage and inflammation, thus leading to functional impairment and poor quality of life, whereas exercise can trigger adaptive responses and facilitate organ recovery, as reflected by altered miRNA expression in damaged organs. This indicates that muscle- and inflammation-related miRNAs may be potential molecular biomarkers for monitoring the progress of exercise-based cancer rehabilitation during cancer survivorship [11]. However, given the heterogeneity of breast cancer biology and its treatment, initial exploratory studies are needed to determine the association between miRNA expression profiles and specific cancer subtypes or cancer treatments.

Based on our previous review [11], the miRNA panel was selected by prioritizing miRNAs with the following characteristics: (1) primary relevance to muscle mass and function, and inflammation in cancer patients, (2) previously reported correlations between physical or physiological parameters and the expression of miRNAs in blood or plasma. Therefore, we selected a panel of muscle-specific (miR-1, miR-133, miR-208, miR-486, and miR-499) and inflammation-related (miR-21, miR-126, miR-146, and miR-155) miRNAs to conduct an exploratory investigation into their associations with breast cancer subtype, treatment, timing, and age during cancer survivorship.

## Methods

### Ethics approval and plasma sample information

This study was performed in accordance with the principles of the Declaration of Helsinki, and the Clinical Research Ethics Committee of University Hospital Galway approved the use of archived human plasma samples (Ref: C.A. 2828). The commercial healthy plasma pool (Lot No. PLP021822AO) was purchased from the AMSBIO company and consisted of plasma from one African American donor and one Hispanic American donor, with an average age of 52 years. All archived plasma samples were obtained from the Cancer Biobank, Discipline of Surgery, University Hospital of Galway. Five healthy archived samples were from healthy controls, with a median age of 52.2 years (range: 51–55 years). The archived samples from breast cancer patients were included if the donors met the following criteria: (1) aged over 18 years, and (2) diagnosed with breast cancer. However, samples were excluded if their donors had (1) breast cancer with rare subtypes or (2) a confirmed history of other cancer in addition to breast cancer. A total of 71 archived samples from breast cancer patients were obtained. These samples came from breast cancer patients aged from 24–88 years, with a median age of 59 years. Among these 71 plasma samples, there were 29 pretreatment samples, collected from patients who did not receive any treatment at the time of sample collection, and 42 post-treatment samples, collected from patients who received cancer treatment at the time of sample collection. Among these samples, 14 were paired samples, in which pretreatment and post-treatment samples were obtained from the same patients. There were also 15 pretreatment samples and 28 post-treatment samples obtained from different patients, which were classified as unpaired samples. Patient characteristics are shown in Table 1.

**Table 1:** Patient characteristics of included samples

Characteristics	Healthy	Pre-treatment	Post-treatment
Number of samples (N)	6	29	42
Age			
Median (year)	52	50	62
< = 50	-	15	12
> 50	-	14	30
Subtypes (N)			
Luminal A	-	15	25
Luminal B	-	5	6
HER2 +	-	5	5
Basal-like	-	4	6
Treatment received			
Surgery	-	-	6
Chemotherapy	-	-	27
Endocrine therapy	-	-	4
Combined therapy	-	-	5

HER2 + : human epidermal growth factor receptor 2 positive.

### RNA isolation

Archived plasma samples were stored at – 80 ℃ and thawed at a 37 ℃ water bath before RNA isolation. Plasma miRNA was isolated using the miRNeasy Serum/Plasma Kit (Qiagen, Cat. No. 217184) according to the manufacturer’s instructions. Plasma aliquots were transferred to new microcentrifuge tubes with five volumes of the QIAzol lysis reagent without spike-ins. RNA concentration was measured using a NanoDrop spectrophotometer (NanoDrop ND-100, Thermo Fisher Scientific, DE, USA).

### miRNA expression by quantitative reverse transcription polymerase chain reaction (qRT-PCR)

cDNA was synthesized using the TaqMan microRNA Reverse Transcription Kit (Thermo Fisher Scentific, Cat. No. 4366597) with 50 ng of RNA in a 15 μL reaction system (RNA: 5 μL, master mix: 7 μL, primer: 3 μL) according to the manufacturer’s instructions. The thermal reaction conditions for miRNA-specific reverse transcription reaction were as follows: 16 ℃ for 30 min, 42 ℃ for 30 min, 85 ℃ for 5 min, and 4 ℃ for hold. All primers were purchased from Thermo Fisher Scentific, and the assay information is provided in Table S1. Quantitative PCR was then performed on a 96-well Fast Thermal Cycling plates (Cat. No. 4346907) using StepOnePlus™ Real-Time PCR System (Applied Biosystems, MA, USA). The amplification conditions were as follows: 50 ℃ for 2 min, 95 ℃ for 10 min, followed by 40 cycles of 95 ℃ for 15 s and 60 ℃ for 1 min. All assays were conducted in triplicate. The cycle threshold (Ct) value was used to calculate the miRNA expression level, and the relative expression levels of selected miRNAs in each plasma sample were normalized to RNU6B and calculated using the 2^ − ΔCt method.

### Statistical analysis

Statistical analyses were performed using SPSS version 25.0 and GraphPad Prism version 8.0. Considering the distribution characteristics of relative miRNA expression levels, miRNA data were log10-transformed for analysis. miRNA expression data were presented with Mean ± standard deviation (SD). The student’s *t-test* or one-way analysis of variance (ANOVA) was performed when data were normally distributed; the Mann–Whitney U test or Kruskal–Wallis test was performed when data were not normally distributed. For multiple-group comparisons, significance values were adjusted by the Bonferroni correction. The paired *t-test* or Wilcoxon signed-rank test was performed for paired-sample analysis. All *p* values were two-sided, and the significance level was set at 0.05.

## Results

### Circulating miRNA profiles in healthy controls and pretreatment patients with breast cancer

We first measured circulating levels of selected muscle-specific and inflammation-related miRNAs in healthy controls, including a healthy plasma pool and five healthy plasma samples, and in pretreatment patients with breast cancer. Muscle-related miRNAs, including miR-1, miR-133, miR-486, and miR-499, were detected at low levels in healthy individuals (Fig. S1), whereas miR-208 was not detected in most samples (data not shown). Comparison of pretreatment patients with breast cancer (n = 29) with healthy controls (n = 6) revealed no statistically significant differences in the expression of any of the selected miRNAs, despite trends toward increased miR-21 and miR-499 levels, and decreased miR-133, miR-486, and miR-155 levels (Table 2). These results indicate that, at the group level, breast cancer alone was not associated with substantial alterations in circulating muscle- or inflammation-related miRNAs before treatment.

**Table 2:** Expression of selected miRNAs in plasma samples from healthy controls and breast cancer patients

	Healthy vs pretreatment				Paired samples				Unpaired samples			
miRNAs	Healthy (N = 6)	Pretreatment (N = 29)	Fold change	P value	Pretreatment (N = 14)	Post-treatment (N = 14)	Fold change	P value	Pretreatment (N = 15)	Post-treatment (N = 28)	Fold change	P value
miR-451	3.05 (0.69)	3.25 (1.33)	1.07	0.718	0.24(1.13)	1.07(1.32)	4.46	0.062	3.21(1.53)	3.66(1.29)	1.14	0.320
miR-1	0.37 (0.85)	0.35 (0.60)	0.95	0.927	-0.31(0.60)	0.12(0.78)	-	0.125	0.46(0.59)	0.53(0.68)	1.15	0.725
miR-21	0.05 (0.43)	0.51 (0.70)	10.2	0.174	0.30(0.70)	1.03(0.4)	3.43	***0.002***	3.20(0.65)	3.14(0.58)	0.98	0.752
miR-126	2.79 (0.62)	3.10 (0.65)	1.11	0.295	0.21(0.66)	0.76(0.73)	3.62	***0.038***	0.59(1.12)	1.17(0.80)	1.98	0.059
miR-133	1.01 (0.81)	0.60 (1.07)	0.59	0.375	-0.40(1.05)	0.26(0.94)	-	0.053	2.97(0.90)	2.81(1.07)	0.95	0.541
miR-146	2.61 (0.68)	2.70 (1.18)	1.03	0.535	-0.21(1.41)	0.53(1.3)	-	0.083	1.36(0.81)	1.27(0.68)	0.93	0.715
miR-155	1.41 (0.67)	1.14 (0.97)	0.81	0.881	-0.51(1.10)	-0.02(0.96)	-	0.152	0.64(0.66)	0.71(0.60)	1.11	0.739
miR-486	3.25 (0.61)	2.80 (1.37)	0.86	0.782	-0.35(1.37)	0.61(1.06)	-	***0.002***	2.70(1.42)	3.51(1.09)	1.30	***0.037***
miR-499	-0.66(0.70)	-0.19(0.85)	3.74	0.342	0.50(1.12)	0.91(0.72)	1.82	0.239	-0.27(0.62)	-0.07(0.64)	-	0.335

*For paired samples, paired t-test or Wilcoxon signed-rank test was applied. For unpaired comparisons, Student’s t-test or Mann–Whitney U test was applied.*

In this study, miR-451 was included as the biomarker for monitoring hemolysis in plasma samples based on the previous literature [12]. Although only clear and transparent plasma samples were used, miR-451 showed significant differences in some analyses. This may be partly attributed to the influence of cancer and cancer therapy on miR-451 [13, 14], suggesting that miR-451 may not be a reliable biomarker of hemolysis in this context. Therefore, the results for miR-451 are presented in the following tables and figures without further interpretation.

### Association between breast cancer subtypes and circulating miRNA expression

**Fig. 1:**
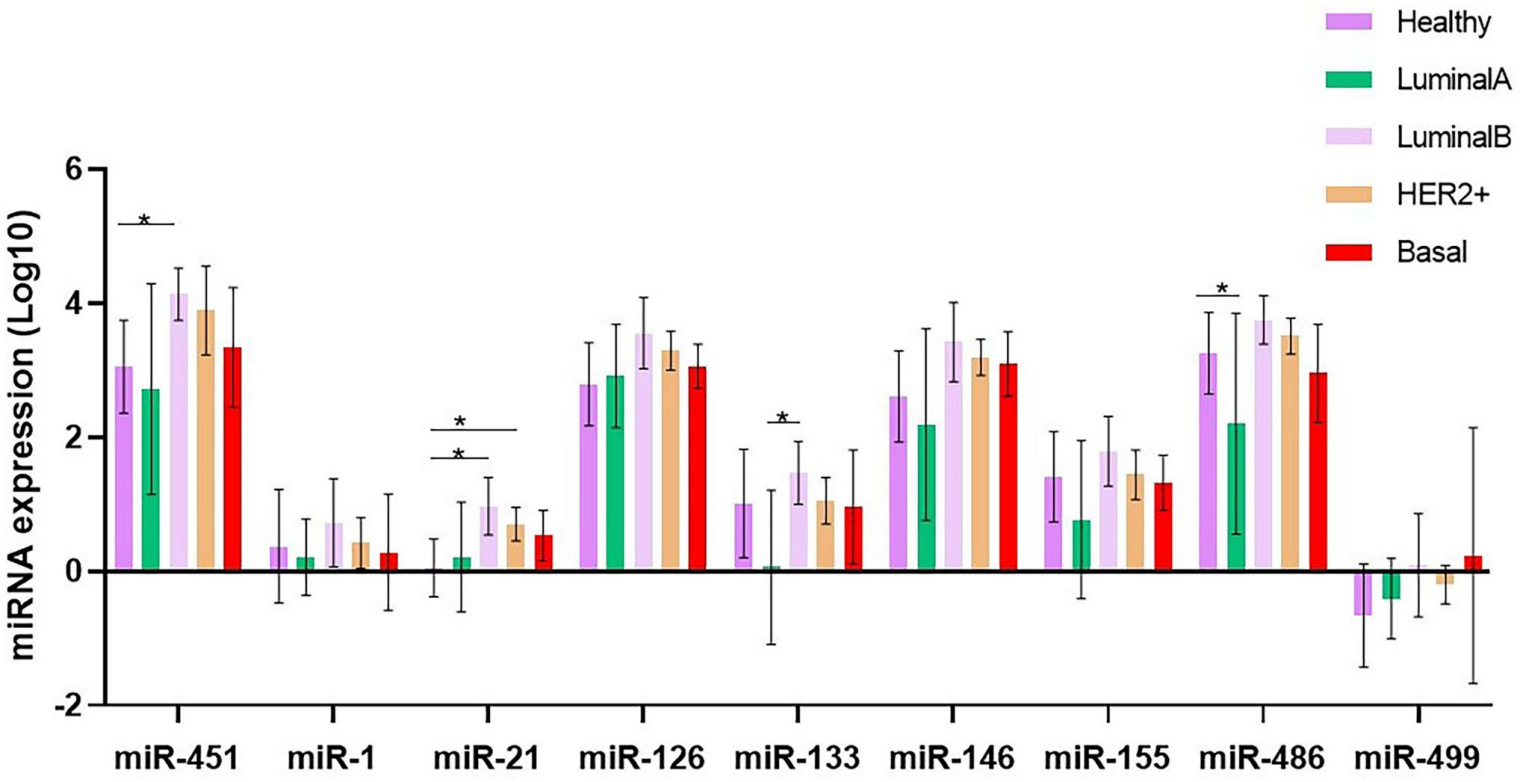
Expression of selected miRNAs across different breast cancer subtypes. The Student’s t-test or Mann–Whitney U test was performed to compare miRNA expression in different cancer subtypes to healthy controls. ANOVA or Kruskal–Wallis test was performed to compare the expression levels of selected miRNAs among different cancer subtypes, p values for within-group comparisons were adjusted using the Bonferroni correction. *represents p < 0.05; **represents p < 0.01; ***represents p < *0.001*

Because breast cancer is biologically diverse, we examined circulating miRNA levels across different molecular subtypes using pre-treatment samples only. Luminal A breast cancer was associated with lower circulating levels of muscle-related miRNAs compared with healthy controls, particularly miR-486 (0.68-fold, *p* = 0.047, Fig. 1, Table S2). miR-133 (0.06-fold, *p* = 0.081) was also lower in patients with Luminal A breast cancer, though this difference did not reach statistical significance. In Luminal B breast cancer, inflammation-related miRNAs were more prominently affected, with increases in miR-21 (19.32-fold, *p* = 0.008), miR-126 (1.27-fold, *p* = 0.062), and miR-146 (1.31-fold, *p* = 0.065). In the human epidermal growth factor receptor 2 positive (HER2 +) subtype, miR-21 (14.10-fold, *p* = 0.042) was significantly increased, but similar trend was not seen in other inflammation-related miRNAs. No significant differences were observed for the basal-like subtype. These findings indicate that muscle- and inflammation-related miRNAs may display subtype-specific expression patterns. However, since samples from the healthy controls in this study were derived from mixed sources and the sample sizes of both control group and each subtype group were relatively small, these results are more descriptive than definitive and should be interpreted with caution.

### Impact of cancer treatment on circulating miRNA expression

To assess the effects of cancer treatment on circulating miRNA levels, we compared post-treatment and pre-treatment samples using both unpaired and paired analyses. In unpaired analyses, post-treatment patients had significantly higher levels of miR-486 than pretreatment patients (Table 2). Paired analyses showed similar results of miR-486. In addition, the paired analyses also revealed significant increases in miR-21 and miR-126 after treatment, which were not seen in unpaired comparisons. Other miRNAs, including miR-133 and miR-146, showed similar trends toward post-treatment changes but were not statistically significant. These results suggest that changes in circulating miRNA profiles are primarily linked to cancer treatment, rather than cancer diagnosis alone.

### Treatment modality–specific miRNA responses

**Fig. 2:**
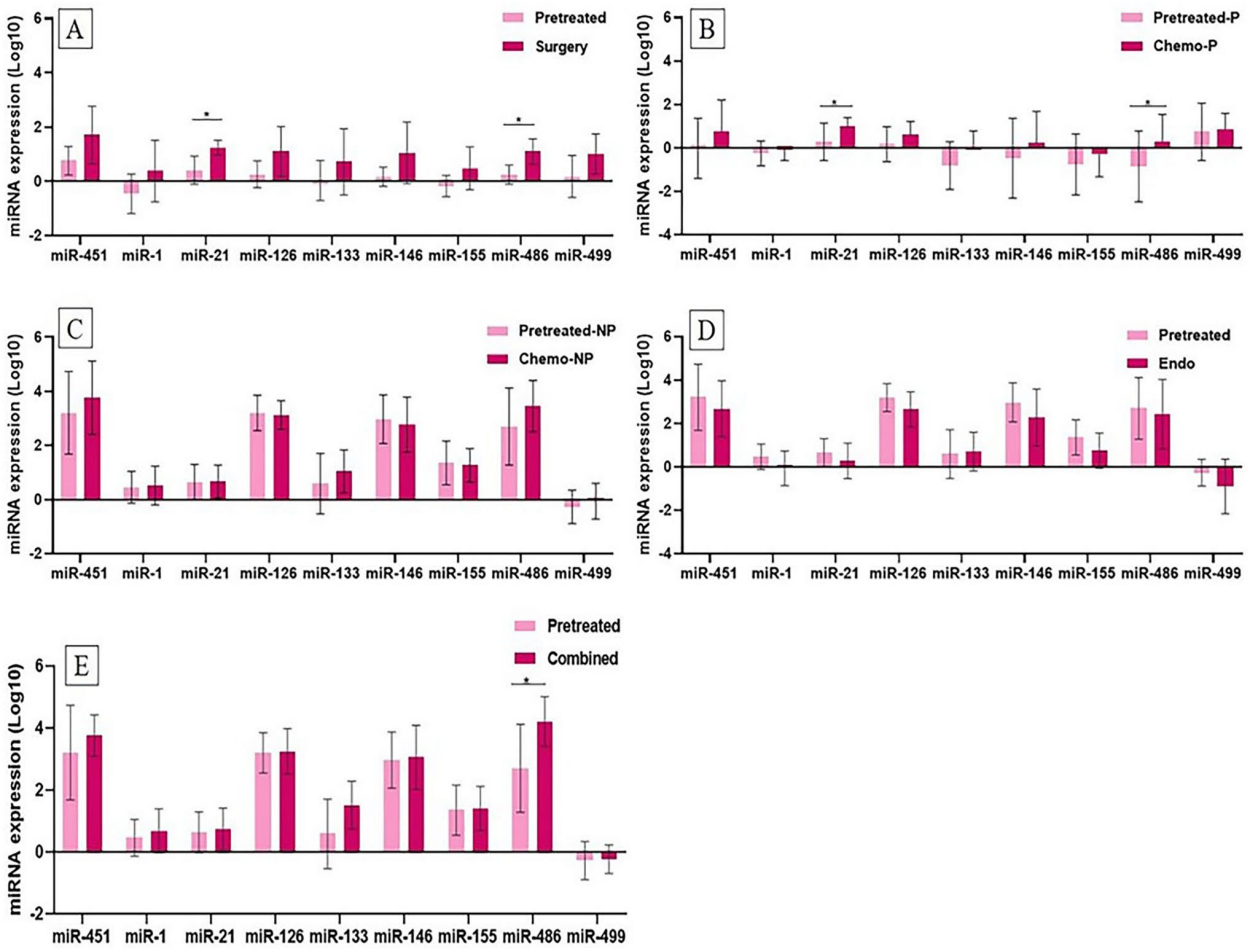
The expression of selected miRNAs in surgery (**A**), chemotherapy (**B**, **C**), endocrine (**D**), and combined therapy (**E**). (**A**)* paired surgery group: 5 pretreatment vs 5 post-treatment; *(**B**)* paired chemotherapy group: 8 vs 8, and *(**C**)*unpaired chemotherapy group (15 vs 19); *(**D**)* endocrine therapy:15 vs 4; *(**E**) combined therapy: 15 vs 5 (one paired), and the paired samples in this group were regarded as independent samples. Paired t-test or Wilcoxon signed-rank test was performed for paired samples; Student’s t-test or Mann–Whitney U test was performed for unpaired samples. *represents p < 0.05; **represents p < 0.01; ***represents p < *0.001*

We then looked at how different treatment modalities affected circulating miRNA levels. Both surgery and chemotherapy were associated with significant increases in miR-21 and miR-486 when pretreatment and post-treatment samples were compared (Fig. 2A-C). These trends were observed in both paired and unpaired analyses, but were more frequently significant in paired comparisons. Endocrine therapy was not associated with significant changes in any of the examined miRNAs (Fig. 2D). In patients receiving combined therapy, which most commonly included surgery and chemotherapy, miR-486 again demonstrated a significant increase compared with pretreatment samples (Fig. 2E). Across treatment modalities, miR-21 exhibited a consistent tendency toward increased expression following treatment exposure, supporting its potential sensitivity to treatment-related physiological changes.

### Subtype-specific treatment-associated miRNA changes

**Fig. 3:**
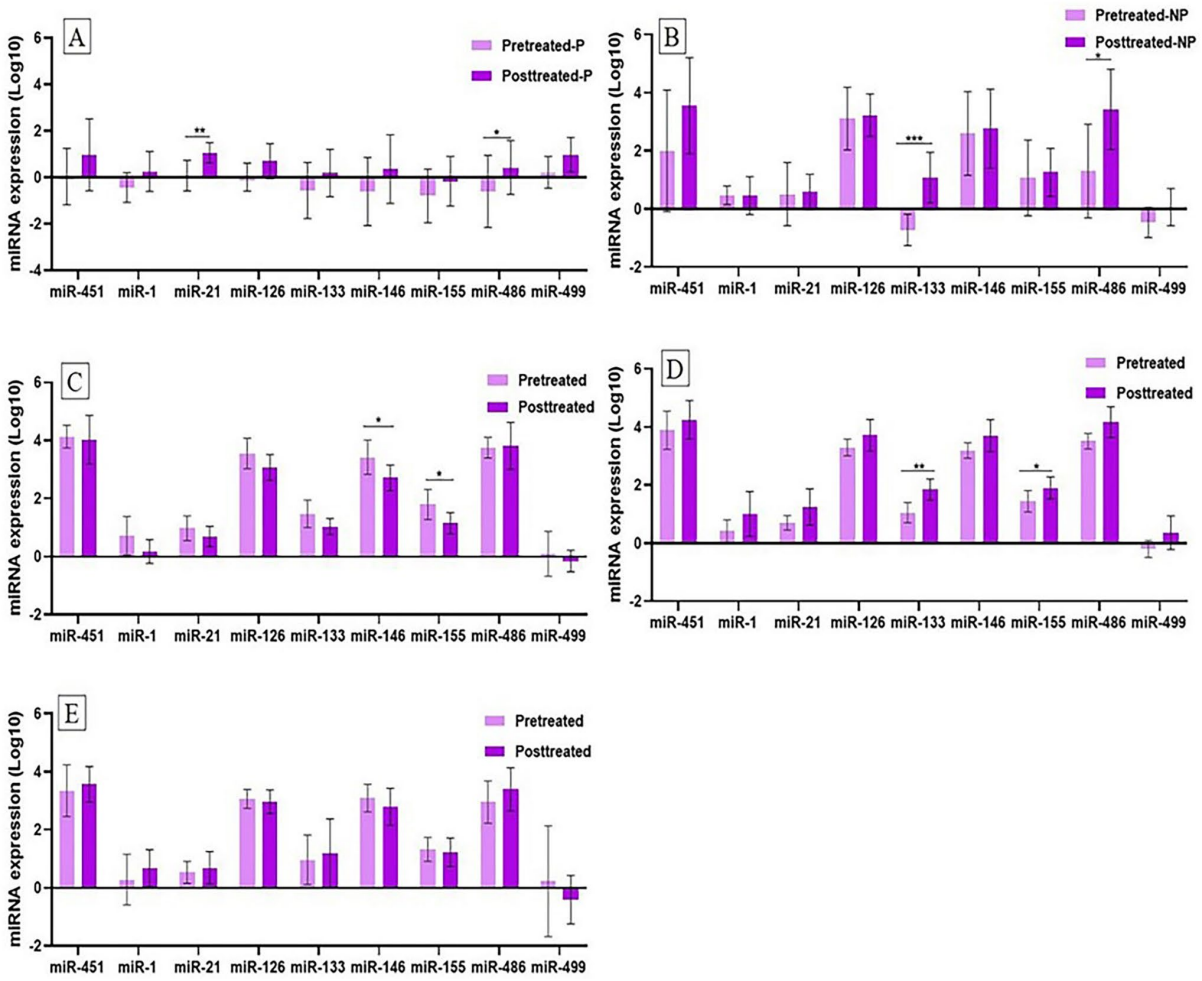
The expression of selected miRNAs in pretreatment and post-treatment groups in Luminal A (**A**, **B**), Luminal B (**C**), HER2 + (**D**), and basal-like (**E**) breast cancer. (**A**)* paired Luminal A: 10 vs 10; *(**B**)* unpaired Luminal A: 5 vs 15; *(**C**)*Luminal B: 5 vs 6 (one paired); *(**D**)* HER2* + *: 5 vs 5 (two paired); *(**E**) Basal-like: 4 vs 6 (1 paired). Due to the small number of paired samples, the paired samples in these groups were regarded as independent samples. Paired t-test or Wilcoxon signed-rank test was performed for paired samples; Student’s t-test or Mann–Whitney U test was performed for unpaired samples. *represents p < 0.05; **represents p < 0.01; ***represents p < *0.001*

We also examined treatment-related miRNA changes within each breast cancer subtype. In Luminal A breast cancer, treatment led to significant increases in miR-486 and miR-21 in paired analyses (Fig. 3A), and an increase in miR-133 in unpaired analyses (Fig. 3B). Although there were some differences between paired and unpaired results, the overall pattern was similar. In Luminal B breast cancer, inflammation-related miRNAs miR-146 and miR-155 demonstrated significant decreases following treatment, despite modest elevations in pretreatment patients relative to healthy controls (Fig. 3C). In HER2 + breast cancer, treatment exposure was associated with increased circulating miR-133 and miR-155 (Fig. 3D). No statistically significant treatment-associated changes were observed in the basal-like subtype (Fig. 3E). These results show that the way circulating miRNA responses to cancer treatment may vary by breast cancer subtype, likely due to differences in tumor biology and treatment approaches.

### Association between treatment duration and circulating miRNA expression

**Fig. 4:**
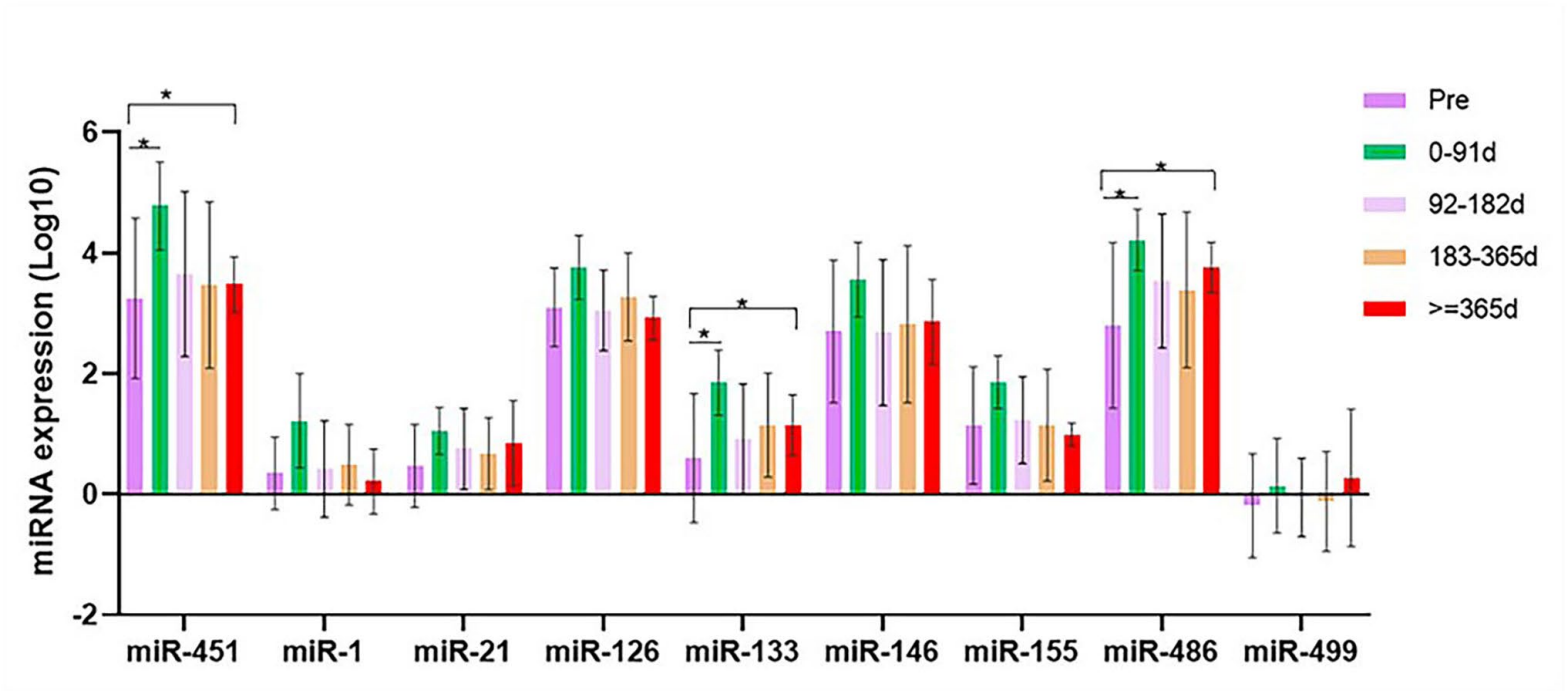
The impact of treatment duration on miRNA change. The bracket represents the p value from ANOVA or the Kruskal–Wallis test; the solid line represents the p value from within-groups comparison, and p values were adjusted by the Bonferroni correction. *represents p < 0.05; **represents p < 0.01; ***represents p < *0.001*

To examine temporal patterns, post-treatment samples were stratified according to the time from diagnosis to blood collection (group information in Table S3). Circulating miRNA changes were most pronounced within the first 91 days after diagnosis (Fig. 4). During this early period, miR-133 and miR-486 were significantly elevated compared with pretreatment samples. After 91 days, circulating miRNA levels mostly returned to pretreatment values, and there were no significant differences at later time points. Most samples collected from the first 91 days were obtained from patients having surgery or neoadjuvant chemotherapy, showing that the early treatment phase may represent a period of greater physiological change.

**Fig. 5:**
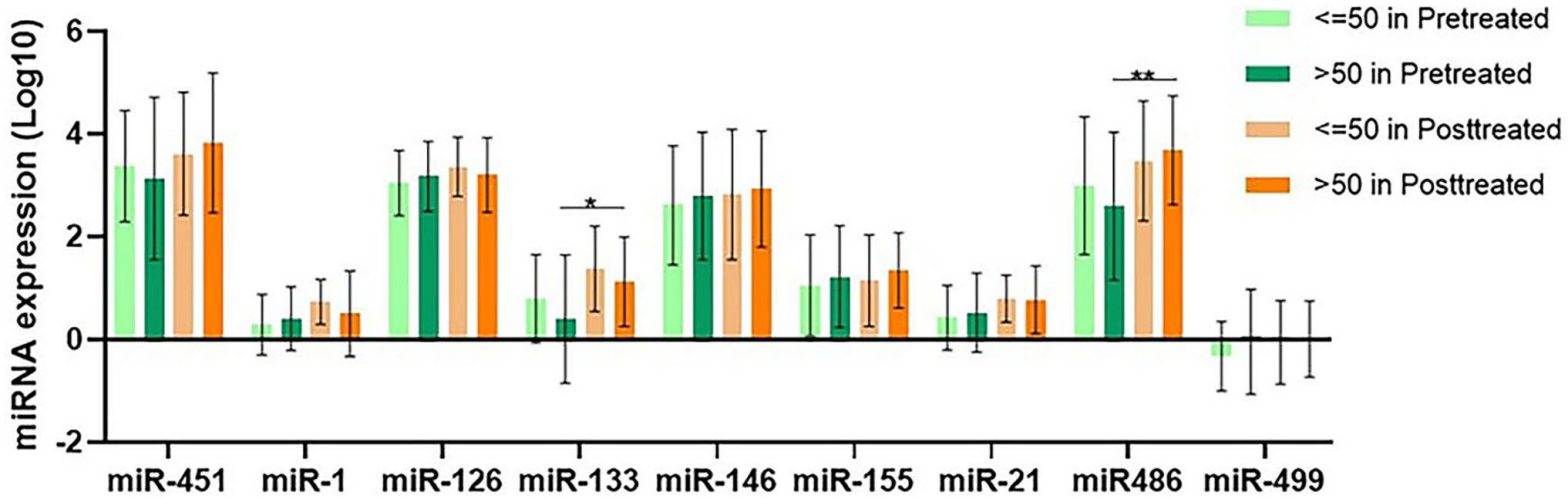
The expression of selected miRNAs in different age groups. Student t-test or Mann–Whitney U test was performed. *represents p < 0.05; **represents p < 0.01; ***represents p < *0.001*

We also examined miRNA levels by age group (age distribution in Table S4). In patients 50 years or younger, there were no significant differences between pretreatment and post-treatment groups (Fig. 5). In patients over 50 years of age, treatment led to significant increases in miR-133 and miR-486. These results suggest that age may influence circulating miRNA responses to cancer treatment, especially for muscle-related miRNAs involved in physical function and recovery.

## Discussion

### Muscle- and inflammation-related miRNAs have different expression patterns among breast cancer subtypes

The expression of miRNA can be influenced by multiple factors. Research has demonstrated that miRNA expression profiles in tumor tissue differ from those in circulation, and that some of the differentially expressed circulating miRNAs are novel, denoting that miRNAs may be selectively released into the circulation. This selective release may be one of the factors affecting miRNA expression in circulation [15, 16]. Similarly, our results showed that muscle-specific miRNAs, including miR-1, miR-133, miR-486, and miR-499, were expressed at low levels in the plasma of healthy controls, which may support the possibility of selective release into circulation. Blenkiron et al. [17] profiled miRNA expression in 93 breast cancer patients and identified 133 differentially expressed miRNAs between healthy breast tissue and breast cancer tissue. Furthermore, miR-155 and miR-146 were differentially expressed among these cancer subtypes, with miR-146 showing higher expression in Luminal A than in Luminal B, indicating that the cancer subtype may influence miRNA expression in breast cancer. However, the findings in our exploratory study were not completely consistent with previous reports, as none of the selected miRNAs were significantly different in plasma of breast cancer patients. When stratified by breast cancer subtypes, muscle-related miRNA miR-486 (*p* = 0.047) and miR-133 (*p* = 0.081, close to significance threshold) were decreased in Luminal A. In contrast, in Luminal B breast cancer patients, inflammation-related miR-21 (*p* = 0.008) was 19-fold higher than that in healthy controls, miR-126 (*p* = 0.062) and miR-146 (*p* = 0.065) increased by approximately 30%. These results revealed that Luminal A may have a greater association with changes in muscle-related miRNAs, while Luminal B may have a greater association with changes in inflammation-related miRNAs, suggesting the potential impact of cancer subtypes on miRNA expression.

### miRNAs have different responses to cancer treatment in breast cancer

Because miRNA signatures differ among breast cancer subtypes and each subtype may receive different treatment strategies, miRNA responses may vary across cancer treatments [18]. In our study, cancer treatment was associated with increased expression of miR-21, miR-126, and miR-486. Compared to pretreatment samples, miR-486 was significantly increased following treatment; however, miR-486 had a decreased expression in post-treatment samples when compared to healthy controls. This may partly reflect treatment-related reversal of cancer-associated alterations. Notably, miR-486 and miR-21 were consistently upregulated in response to surgery and chemotherapy and in Luminal A breast cancer, suggesting their potential relevance in patients with this subtype. Previous research has shown that miR-21 is associated with the response to HER2-targeted therapy or the modulation of HER2 expression [18], but this was not observed in our results. Additionally, miR-126 was significantly increased after cancer treatment in paired analyses, but did not present any significance in the subgroup analyses. Therefore, the potential relevance of miR-126 should be carefully interpreted. miR-146 and miR-155 were significantly decreased in Luminal B breast cancer following cancer treatment, while miR-133 was significantly increased in Luminal A and HER2 + breast cancer following treatment. These results may imply that the changes in miRNAs were likely to be treatment modality-specific or subtype-specific. However, given the small sample size in each subgroup, the findings should be interpreted cautiously, and confirmatory studies with larger sample sizes are needed.

### Implications of miR-486, miR-133, and miR-21 during cancer survivorship

Plasma miR-486 levels were generally lower in breast cancer patients than in healthy controls, but elevated following cancer treatment in our study. This pattern aligns with previous reports in mouse models and breast cancer patients [19, 20]. However, it contradicts previous findings in cervical cancer and non-small cell lung cancer (NSCLC), where circulating miR-486 levels in NSCLC and cervical cancer were higher in cancer patients than in healthy controls [21, 22], suggesting that the expression of plasma miR-486 may vary across cancer types. Notably, in NSCLC, plasma miR-486 levels significantly increased after surgical resection, and this increase persisted up to one year after surgery [21], a trend that parallels our own observations in breast cancer and highlights the potential broad relevance of this biomarker. Considering the known role of miR-486 in muscle development and function maintenance, our findings, together with previous evidence, indicate that circulating miR-486 is dynamic and context-dependent—varying with cancer type—and may serve as a potential biomarker of muscle damage or turnover in cancer survivorship.

In this study, breast cancer itself did not alter the expression of miR-133, which is inconsistent with previous reports, where miR-133 was reported to be downregulated in ovarian cancer and breast cancer [23, 24]. Moreover, miR-133 did not differ between pretreatment and post-treatment samples in the unpaired analysis; however, the paired analysis approached statistical significance, indicating that inter-individual variability may have obscured the treatment effect. Importantly, subgroup analyses revealed a significant increase in miR-133 specifically in Luminal A and HER2 + breast cancer following cancer treatment. These results further suggest that the overall limited sensitivity of miR-133 as a biomarker may be attributed to both sample variability and differential responses across cancer subtypes. Research has shown that serum miR-133 is significantly elevated in acute myeloid leukemia following standard induction chemotherapy, especially in those with a favorable response or complete remission [25]. Notably, changes in both miR-133 and miR-486 expression were most pronounced within the first 91 days and after 365 days post-diagnosis, respectively, indicating a time-specific pattern. Moreover, these alterations were particularly evident in patients aged over 50 years, suggesting an age-dependent susceptibility.

The expression patten of miR-21 following cancer treatment remains controversial. In our study, miR-21 was significantly higher in breast cancer patients, especially in Luminal B and HER2 + , than in healthy controls, and increased further after surgery and chemotherapy. Similarly, Muller et al. [26] reported higher expression levels of serum miR-21 in HER2 + breast cancer compared to healthy controls, and miR-21 increased further after chemotherapy combined with either trastuzumab or lapatinib [26]. In contrast, Khalighfard et al. [27] found that plasma miR-21 was significantly increased in Luminal A breast cancer patients, but it was significantly down-regulated after surgery, chemotherapy, and radiotherapy compared to pretreatment levels. These results again underscored that miR-21 may be treatment modality-specific and subtype-specific.

## Conclusion

This exploratory study shows circulating muscle- and inflammation-related miRNAs display distinct expression patterns associated with breast cancer subtype, treatment modalities, timing, and patient age. Specifically, miR-21, miR-133, and miR-486 demonstrate sensitivity to cancer treatment exposure, timing, and patient age, suggesting their potential as circulating biomarkers for monitoring inflammation- and muscle-related changes during breast cancer survivorship. These miRNAs have previously been investigated in cellular and animal models, as well as retrospective and prospective clinical studies, where their functional roles have been reported. Therefore, our study focused on evaluating their clinical relevance as circulating biomarkers in breast cancer survivors rather than re-establishing their biological functions.

However, this study has some limitations, including its retrospective design, relatively small subgroup sizes, and lack of physical function and recovery measures. As an initial exploratory investigation, this study aimed to identify circulating miRNAs that may serve as candidate biomarkers for monitoring rehabilitation outcomes as our research program is primarily centered on exercise-based cancer rehabilitation during survivorship. Given the biological heterogeneity of breast cancer and the potential influence of different treatment modalities, we considered it important first to establish associations between circulating miRNA expression and clinical phenotypes before pursuing mechanistic studies.

Future studies should be conducted in a larger cohort and include measures of muscular and inflammatory indicators to determine whether these miRNAs can help monitor adaptation and guide exercise-based rehabilitation in breast cancer survivors. We acknowledge that functional validation, such as gain- or loss-of-function experiments using miRNA mimics or inhibitors in relevant muscle or cancer cell models, and ultimately in vivo studies, will be essential to elucidate the biological mechanisms underlying these associations. These experiments are beyond the scope of the present exploratory study but constitute an important direction for our future research. The findings reported here provide a foundation for selecting the most promising candidate miRNAs for such mechanistic investigations.

## Supplementary Information

Below is the link to the electronic supplementary material.

Supplementary file1 (DOCX 58 KB)

## Data Availability

Data supporting the findings of this study are available in supplementary files.

## References

[1] Harbeck N et al (2019) Breast cancer. Nat Rev Dis Prim 5(1):1–31. 10.1038/s41572-019-0111-2 31548545 10.1038/s41572-019-0111-2

[2] Bray BF et al (2022) Global cancer statistics 2022: GLOBOCAN estimates of incidence and mortality worldwide for 36 cancers in 185 countries. CA Cancer J Clin 74(3):229–263. 10.3322/CAAC.21834 10.3322/caac.21834 38572751

[3] Jang MK, Park S, Raszewski R, Park CG, Doorenbos AZ, Kim S (2024) “Prevalence and clinical implications of sarcopenia in breast cancer: a systematic review and meta-analysis.” Supportive Care Cancer 32(5):328. 10.1007/S00520-024-08532-0 10.1007/s00520-024-08532-0 38702479

[4] Villaseñor A et al (2012) Prevalence and prognostic effect of sarcopenia in breast cancer survivors: the HEAL study. J Cancer Surviv 6(4):398–406. 10.1007/S11764-012-0234-X 23054848 10.1007/s11764-012-0234-x PMC3747827

[5] Onesti JK, Guttridge DC (2014) Inflammation based regulation of cancer cachexia. BioMed Res Int. 10.1155/2014/168407 24877061 10.1155/2014/168407 PMC4022077

[6] Diakos CI, Charles KA, McMillan DC, Clarke SJ (2014) Cancer-related inflammation and treatment effectiveness. Lancet Oncol 15(11):e493–e503. 10.1016/S1470-2045(14)70263-3 25281468 10.1016/S1470-2045(14)70263-3

[7] Pérez-Baos S, Prieto-Potin I, Román-Blas JA, Sánchez-Pernaute O, Largo R, Herrero-Beaumont G (2018) Mediators and patterns of muscle loss in chronic systemic inflammation. Front Physiol. 10.3389/FPHYS.2018.00409/BIBTEX 29740336 10.3389/fphys.2018.00409 PMC5928215

[8] Santos JMO, Da Silva SP, Gil Da Costa RM, Medeiros R (2020) The emerging role of micrornas and other non-coding rnas in cancer cachexia. Cancers (Basel) 12(4):1–14. 10.3390/cancers12041004 10.3390/cancers12041004 PMC7226600 32325796

[9] Freire PP et al (2019) The pathway to cancer cachexia: MicroRNA-regulated networks in muscle wasting based on integrative meta-analysis. Int J Mol Sci. 10.3390/IJMS20081962 31013615 10.3390/ijms20081962 PMC6515458

[10] Narasimhan A et al (2017) Small RNAome profiling from human skeletal muscle: novel miRNAs and their targets associated with cancer cachexia. J Cachexia Sarcopenia Muscle 8(3):405–416. 10.1002/jcsm.12168 28058815 10.1002/jcsm.12168 PMC5476855

[11] Jiang Y, Ghias K, Gupta S, Gupta A (2021) MicroRNAs as potential biomarkers for exercise-based cancer rehabilitation in cancer survivors. Life. 10.3390/LIFE11121439 34947970 10.3390/life11121439 PMC8707107

[12] Kirschner MB et al (2011) Haemolysis during sample preparation alters microRNA content of plasma. PLoS ONE 6(9):e24145. 10.1371/JOURNAL.PONE.0024145 21909417 10.1371/journal.pone.0024145 PMC3164711

[13] Pan X, Wang R, Wang ZX (2013) The potential role of miR-451 in cancer diagnosis, prognosis, and therapy. Mol Cancer Ther 12(7):1153–1162. 10.1158/1535-7163.MCT-12-0802/93940/P/THE-POTENTIAL-ROLE-OF-MIR-451-IN-CANCER-DIAGNOSIS 23814177 10.1158/1535-7163.MCT-12-0802

[14] Woo JW, Choi HY, Kim M, Chung YR, Park SY (2022) miR-145, miR-205 and miR-451: potential tumor suppressors involved in the progression of in situ to invasive carcinoma of the breast. Breast Cancer 29(5):814–824. 10.1007/S12282-022-01359-9/FIGURES/4 35451796 10.1007/s12282-022-01359-9

[15] Zhu J et al (2014) Different miRNA expression profiles between human breast cancer tumors and serum. Front Genet. 10.3389/FGENE.2014.00149/ABSTRACT 24904649 10.3389/fgene.2014.00149 PMC4033838

[16] Chan M et al (2013) Identification of circulating microRNA signatures for breast cancer detection. Clin Cancer Res 19(16):4477–4487. 10.1158/1078-0432.CCR-12-3401/85841/AM/IDENTIFICATION-OF-CIRCULATING-MICRORNA-SIGNATURES 23797906 10.1158/1078-0432.CCR-12-3401

[17] Blenkiron C et al (2007) MicroRNA expression profiling of human breast cancer identifies new markers of tumor subtype. Genome Biol 8(10):1–16. 10.1186/GB-2007-8-10-R214/FIGURES/6 10.1186/gb-2007-8-10-r214 PMC2246288 17922911

[18] Andorfer CA, Necela BM, Thompson EA, Perez EA (2011) MicroRNA signatures: clinical biomarkers for the diagnosis and treatment of breast cancer. Trends Mol Med 17(6):313–319. 10.1016/J.MOLMED.2011.01.006 21376668 10.1016/j.molmed.2011.01.006

[19] Wang R et al (2022) Skeletal muscle-specific overexpression of miR-486 limits mammary tumor-induced skeletal muscle functional limitations. Mol Ther Nucleic Acids 28:231–248. 10.1016/J.OMTN.2022.03.009 35402076 10.1016/j.omtn.2022.03.009 PMC8971682

[20] Rask L et al (2014) Differential expression of miR-139, miR-486 and miR-21 in breast cancer patients sub-classified according to lymph node status. Cell Oncol 37(3):215–227. 10.1007/S13402-014-0176-6/FIGURES/5 10.1007/s13402-014-0176-6 PMC13004441 25027758

[21] Sromek M et al (2017) Changes in plasma miR-9, miR-16, miR-205 and miR-486 levels after non-small cell lung cancer resection. Cell Oncol 40(5):529–536. 10.1007/S13402-017-0334-8/FIGURES/4 10.1007/s13402-017-0334-8 PMC13001572 28634901

[22] Li C, Zheng X, Li W, Bai F, Lyu J, Meng QH (2018) Serum miR-486-5p as a diagnostic marker in cervical cancer: With investigation of potential mechanisms. BMC Cancer 18(1):1–10. 10.1186/S12885-017-3753-Z/FIGURES/7 29316891 10.1186/s12885-017-3753-z PMC5759341

[23] Zhou Y, Cheng Z (2022) The roles of microRNA-133 in gynecological tumors. Gynecol Minim Invasive Ther 11(2):83. 10.4103/GMIT.GMIT_79_20 35746911 10.4103/GMIT.GMIT_79_20 PMC9212183

[24] Hesari AR et al (2019) Expression of circulating miR-17, miR-25, and miR-133 in breast cancer patients. J Cell Biochem 120(5):7109–7114. 10.1002/JCB.27984 30485486 10.1002/jcb.27984

[25] Zheng ZZ et al (2020) Serum miR-133 as a novel biomarker for predicting treatment response and survival in acute myeloid leukemia. Eur Rev Med Pharmacol Sci 24(2):777–782. 10.26355/EURREV_202001_20060 32016982 10.26355/eurrev_202001_20060

[26] Müller V et al (2014) Changes in serum levels of miR-21, miR-210, and miR-373 in HER2-positive breast cancer patients undergoing neoadjuvant therapy: A translational research project within the Geparquinto trial. Breast Cancer Res Treat 147(1):61–68. 10.1007/S10549-014-3079-3/FIGURES/3 25086636 10.1007/s10549-014-3079-3

[27] Khalighfard S, Alizadeh AM, Irani S, Omranipour R (2018) Plasma miR-21, miR-155, miR-10b, and Let-7a as the potential biomarkers for the monitoring of breast cancer patients. Scientific Rep. 10.1038/s41598-018-36321-3 10.1038/s41598-018-36321-3 PMC6299272 30568292

