# Supplementary material for: Circulating muscle- and inflammation-related microRNAs in breast cancer survivorship: associations with subtype, treatment, timing, and age
Source: Mol Biol Rep. 2026 Jul 28;53(1):1287. doi: 10.1007/s11033-026-12472-9 (PMC13415333; doi:10.1007/s11033-026-12472-9)
Supplement: Supplementary file 1 — Supplementary file1 (DOCX 58 KB) [file 11033_2026_12472_MOESM1_ESM.docx]

Supplementary Tables and Figures

Supplementary Table 1 The sequence of miRNA primers

| miRs | miRbase ID | Assay ID | miRNA Sequence |
| --- | --- | --- | --- |
| RNU6B | 568915 | 001093 | CGCAAGGATGACACGCAAATTCGTGAAGCGTTCCATATTTTT |
| miR-451 | hsa-miR-451a | 001141 | AAACCGUUACCAUUACUGAGUU |
| miR-1 | hsa-miR-1-3p | 002222 | UGGAAUGUAAAGAAGUAUGUAU |
| miR-21 | hsa-miR-21-3p | 002438 | CAACACCAGUCGAUGGGCUGU |
| miR-126 | hsa-miR-126-3p | 002228 | UCGUACCGUGAGUAAUAAUGCG |
| miR-133 | hsa-miR-133a-3p | 002246 | UUUGGUCCCCUUCAACCAGCUG |
| miR-146 | hsa-miR-146a-5p | 000468 | UGAGAACUGAAUUCCAUGGGUU |
| miR-155 | hsa-miR-155-5p | 467534-mat | UUAAUGCUAAUCGUGAUAGGGGUU |
| miR-486 | hsa-miR-486-5p | 001278 | UCCUGUACUGAGCUGCCCCGAG |
| miR-499 | hsa-miR-499a-5p | 001253 | UUAAGACUUGCAGUGAUGUUU |

Supplementary Table 2 The expression of selected miRNAs in different cancer subtypes

| miRNA | Healthy | Luminal A | | | Luminal B | | | HER2+ | | | Basal | | |
| --- | --- | --- | --- | --- | --- | --- | --- | --- | --- | --- | --- | --- | --- |
|  | Mean (SD) | Mean (SD) | Fold change | P | Mean (SD) | Fold change | P | Mean (SD) | Fold change | P | Mean (SD) | Fold change | P |
| miR-451 | 3.05 (0.69) | 2.72 (1.57) | 0.89 | 0.634 | 4.13 (0.39) | 1.36 | ***0.012*** | 3.89 (0.66) | 1.28 | 0.115 | 3.34 (0.89) | 1.09 | 0.570 |
| miR-1 | 0.37 (0.85) | 0.21 (0.57) | 0.58 | 0.636 | 0.72 (0.66) | 1.94 | 0.493 | 0.42 (0.38) | 1.13 | 0.833 | 0.28 (0.87) | 0.75 | 0.872 |
| miR-21 | 0.05 (0.43) | 0.21 (0.82) | 4.21 | 0.682 | 0.97 (0.43) | 19.32 | ***0.008*** | 0.70 (0.25) | 14.10 | ***0.042*** | 0.53 (0.38) | 10.69 | 0.118 |
| miR-126 | 2.79 (0.62) | 2.91 (0.77) | 1.04 | 0.758 | 3.55 (0.53) | 1.27 | 0.062 | 3.29 (0.29) | 1.18 | 0.203 | 3.06 (0.33) | 1.10 | 0.459 |
| miR-133 | 1.01 (0.81) | 0.06 (1.15) | 0.06 | 0.081 | 1.47 (0.47) | 1.45 | 0.297 | 1.05 (0.35) | 1.04 | 0.855 | 0.96 (0.85) | 0.95 | 0.927 |
| miR-146 | 2.61 (0.68) | 2.19 (1.43) | 0.84 | 0.503 | 3.42 (0.59) | 1.31 | 0.065 | 3.19 (0.27) | 1.22 | 0.196 | 3.09 (0.48) | 1.18 | 0.258 |
| miR-155 | 1.41 (0.67) | 0.77 (1.18) | 0.54 | 0.228 | 1.79 (0.52) | 1.27 | 0.326 | 1.44 (0.37) | 1.02 | 0.778 | 1.32 (0.41) | 0.94 | 0.815 |
| miR-486 | 3.25 (0.61) | 2.20 (1.65) | 0.68 | ***0.047*** | 3.75 (0.36) | 1.15 | 0.143 | 3.51 (0.27) | 1.08 | 0.507 | 2.95 (0.73) | 0.91 | 0.494 |
| miR-499 | -0.66 (0.70) | -0.41 (0.60) | - | 0.252 | 0.09 (0.77) | -- | 0.106 | -0.20 (0.29) | - | 0.146 | 0.23 (1.91) | -- | 0.338 |

*The expression of target miRNA was normalized by RNU6B and log10-transformed. Data were presented mean±SD; Fold change was calculated by healthy controls and the comparison of selected miRNA was performed by Mann-Whitney test or Student’s t test.*

Supplementary Table 3 The distribution of treatment duration (day)

| Group | N | Median | Minimum | Maximum |
| --- | --- | --- | --- | --- |
| Pre | 29 | 7.00 | -15 | 50 |
| 0-91 | 7 | 35.00 | 28 | 91 |
| 92-182 | 16 | 147.00 | 109 | 182 |
| 183-365 | 16 | 199.50 | 187 | 343 |
| >365 | 3 | 707.00 | 700 | 2112 |
| Total | 71 | 91.00 | -15 | 2112 |

*Note: Treatment duration for each sample was calculated from the day of diagnosis to the day of blood sampling as the exact day of treatment was not able to be known from the patient’s data. A negative day in this table means the sampling date was ahead of the diagnosis date.*

Supplementary Table 4 The age distribution in the pretreatment and post-treatment groups

|  | Age (year) | N | Median | Minimum | Maximum |
| --- | --- | --- | --- | --- | --- |
| Pretreatment | <=50 | 15 | 44.00 | 35 | 50 |
|  | >50 | 14 | 74.00 | 51 | 86 |
| Post-treatment | <=50 | 12 | 45.50 | 24 | 50 |
|  | >50 | 30 | 74.50 | 51 | 88 |
|  | Total | 71 | 59.00 | 24 | 88 |


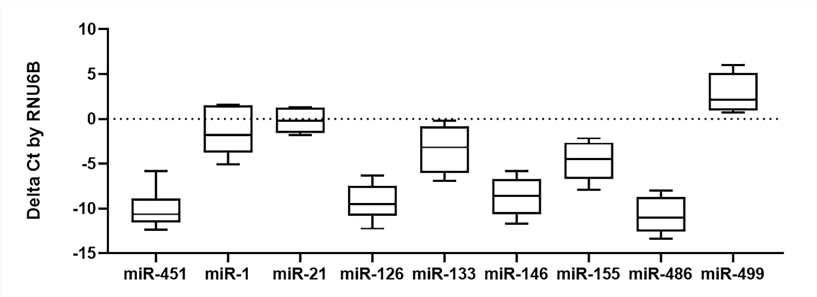


Supplementary Figure 1 The expression of selected miRNAs in healthy controls

*Each miRNA for each sample was performed in three triplicates. The Delta Ct value of each sample was calculated by RNU6B. Data were presented as Mean±SD.*
